# A Contribution to Laryngology

**Published:** 1877-11

**Authors:** E. Cutter

**Affiliations:** Boston, Mass., 13 Temple St., Cambridge, Mass.


### A CONTRIBUTION TO LARYNGOLOGY. — TWO CASES OF DISEASED ARYTENOID CARTILAGE TREATED BY LOCAL DEPLETION.

By E. Cutter, M. D., Cambridge, Mass.

Case I.—A middle aged lady, residing in Newton, Mass., complained of hoarseness, partial loss of voice, dysphagia, cough and throat distress. There was no difficulty in breathing. She was placed under the writer's charge, for examination and treatment, by Prof. Field; and, on inspection with the mirror, the larynx was found to be normal, except that there was a large, red, pear-shaped enlargement of the tip of the left arytenoid cartilage. The right cartilage was of normal size. The left was of the size of a cranberry, or more definitely, from $\frac{1}{2}$ to $\frac{5}{8}$ of an inch in diameter. To the touch it felt hard, but not stony, and was movable from side to side. The question of malignancy was raised, but not sustained by the rational signs. It was, therefore, concluded that the enlargement was not carcinomatous, but hypertrophic; and the subsequent history has sustained the conclusion, as ten years have now elapsed since the patient's recovery.

Satisfied as to the character of the growth, the next question was that of treatment. The writer had then never seen, heard nor read of such a case. The entire subject of laryngology was then in its infancy; and, as a consequence, having no guide, either from experience or history, the treatment was conducted on general principles.

Hypertrophy of the periarytenoid tissues, accompanied by congestion of the blood vessels, involves an enlarged and dilated condition of the capillaries, by blood accumulation, resulting in an altered condition of nutrition, the vessels being, as it were, paralyzed by mechanical distension. By dividing the vessels they would be enabled to unload themselves of their contents and to contract to their normal caliber. Thus they should regain their tonicity and subsequently proceed to a better discharge of their functions. Whether this reasoning were right or wrong, it became really a part of the history of the case, as explaining the course that was pursued. A knife was specially constructed, by Messrs. Codman & Shurtleff, of stout, flattened steel wire. The end was sharpened, like a lancet, on both edges. Two inches from the distal extremity, it formed an angle of 120°. When in position, the flattening was lateral, so that the greatest strength of resistance was in the direction of the thrust. The operation of local depletion was practiced two or three times a week for two months, as follows: the patient was seated as in ordinary laryngoscopy, and the throat was illuminated by natural or artificial light. The parts being exposed to view by the laryngoscope, the special scarificator was then introduced, guided by the eye. Incisions were made freely over and into the substance of the swelling, until the hemorrhage amounted to a mouthful or more. The evacuation of the blood was followed by considerable relief to the local symptoms and subsidence of the enlargement, resulting in the complete relief of the patient.

Since this paper was written, Prof. Field has informed the writer, that the disease had not recurred, and the cure was permanent.

Remarks.—The general tendency of medical opinion is against depletion ; but it is surely an error to desist from its use in chronic engorgements and infarctions. In the present instance, it not only did no harm, but was followed by the happiest results. It should be stated that no pus or serum was discharged, but only blood, and no abscess resulted. The writer has been in the habit of scarifying every local chronic congestion in the throat, and has never yet seen any reason to regret such a course, for incisions of this sort heal very readily, and result in relief from pain. They do good, apparently, by emptying engorged vessels, by relieving the semi-paralyzed condition of their walls, and by allowing them to resume their functions. In the condition of natural tonicity, a capillary will admit, at a time, but one red blood corpuscle, though when congested, it may admit many. When long distended it ceases to respond to such external stimuli as the topical application in common use. It cannot contract, because it is weakened by distension. Divide the vessel, permit the escape of its contents and contraction becomes possible. Its distension removed, the vessel is enabled to perform its normal functions and becomes obedient to those natural laws of the body whereby tissue material is deposited and taken up, so that the symmetry of each organ is not altered.

Case II.—Left arytenoid cartilage enlarged, solid and mobile. Sleep rendered impossible by a sensation of suffocation. Tracheotomy. Removal of tube. Death.

A medium-sized, robust Irishman, 24 years of age, complained of his throat. He had no pain, but suffered from a dry, irritating, convulsive cough, altered voice, dyspnœa, dysphagia, and complete suffocation on falling asleep. The only source to which he could ascribe the trouble was a severe cold contracted a month or more before consultation with the writer. The loss of sleep was the most prominent difficulty; for although he could fall asleep at any time, this condition would be soon disturbed by the sudden occurrence of a sense of complete suffocation, and the patient would be aroused by the efforts put forth to restore respiration.

His general health had not suffered much impairment. Phonation was peculiar, its tone being coarse, gruff and muffled. Inspiration was noisy; expiration noiseless. On laryngoscopic examination, the throat was found to be injected and reddened. Behind the tongue, on the left side, was a whitish globular mass, covered with thin mucous membrane, the tissue beneath resembling cartilage, while the surface was unbroken, smooth and even. This tumor was about as large as an ordinary marble. It was attached below and touched the posterior pharyngeal wall and the back part of the tougue, overhanging the larynx from the left side, so that it was difficult to see the vocal cords, which were found to be intact and normal. The tumor was mobile. When pressed upon, it would wabble over onto the larynx and stop it as completely as if by a ball-valve.

The right arytenoid cartilage was normal; the left, lost in the tumor. Judging from appearances, after a careful examination, it was decided that the growth resulted from an enlargement of the arytenoid cartilage, and this opinion was confirmed by several other physicians who examined it. It was also thought to be carcinomatous, for the following reasons:

1. It so much resembled a case of schirrus of the skin that occurrred in the practice of my late honored father, Dr. Benjamin Cutter. The patient I refer to was a married female, about 50 years of age, suffering from undoubted cancer of the uterus. Beneath the integument of the thorax and abdomen were ten or twelve hard, globular, whitish, movable bodies exactly resembling white marbles.

2. A case occurred in the writer's own practice where similar tumors were developed upon the scalp, which the patient called "horns," as they felt very much like the rudimentary horns of calves. After death it was found that they penetrated both tables of the skull, leaving, after removal, circular openings, extending to the dura mater, as clean cut as if made with a trephine.

In the way of treatment it was decided to try the effects of scarification, encouraged by the good results obtained in the first case. This was faithfully employed. But it was difficult to obtain a mouthful of blood at any sitting owing to the stony hardness and mobility of the tumor, all efforts proving a failure. As a result, the patient's general health began to fail. He grew weak, thin, and nervous from want of sleep, and, finally, could hardly keep awake long enough to permit of the scarification. But no sleep was possible for him, as the consequent and complete closure of his larynx would not permit of it. Fearing that this continual loss of sleep would result fatally, counsel was had, and it was decided to perform tracheotomy in order that the tumor might be allowed to remain at rest, some sleep be secured and a chance for recovery be eventually afforded by quieting the irritation of the larynx. The operation was done with immediate relief and proved to be a critical one. The writer was over-ruled in the choice of an anæsthetic, believing, as he did, that ether would produce such a flooding of the mouth with the secretions of the salivary, parotid and other glands of the buccal cavity, that suffocation would result when insensibility occurred. The objection to the chloroform, however, was so strong that the patient was etherized, and what had been feared immediately resulted. Respiration and cardiac pulsation instantly ceased, and the body become as rigid as a cadaver. It became necessary to make the section of the skin, fascia, muscles, and cartilages of the trachea while feeling at the bottom of a well of blood, regardless of nicety of dissection and hemorrhage. A metal tube was soon inserted into the trachea and rapidly filled with blood. By means of an India rubber tube attached to a syringe bulb, the blood was removed and respiration reestablished. The patient rapidly recovered, lost his anxious countenance and enjoyed a quiet and refreshing sleep.

The effect upon the tumor of the artificial opening, was absolutely nil. While it did not increase, it certainly did not diminish in size. In order to obtain a change of air the patient subsequently removed to Ohio, and while there he consulted some one who told him that if he had been properly treated at first, he might have been cured. In accordance with the advice then given the tube was subsequently removed as useless ! In a short time the man choked to death.

*Remarks.* These two cases illustrate a common experience; of two patients similarly affected and similarly treated, one is relieved and the other not. Both were treated ten years ago. At the present time should I have occasion to treat a patient affected as in the last instance reported, it is probable that I would attempt to remove the growth, after tracheotomy, by the galvano-caustic wire.

BOSTON, Mass., 13 Temple St., July 31st, 1877.

